# Microscopic analysis of metal matrix composites containing carbon Nanomaterials

**DOI:** 10.1186/s42649-019-0024-2

**Published:** 2020-02-10

**Authors:** Daeyoung Kim, Hye Jung Chang, Hyunjoo Choi

**Affiliations:** 1 School of Advanced Materials Engineering, Kookmin University, 02707 Seoul, Republic of Korea; 2 Advanced Analysis Center, Korea Institute of Science and Technology, 02792 Seoul, Republic of Korea; 3 Division of Nano & Information Technology, KIST School, University of Science and Technology, Seoul, 02792 Republic of Korea

**Keywords:** Composites, Carbon nanomaterials, Distribution, Interface, Microstructure

## Abstract

Metallic matrix composites reinforced with carbon nanomaterials continue to attract interest because of their excellent mechanical, thermal, and electrical properties. However, two critical issues have limited their commercialization. Uniform distribution of carbon nanomaterials in metallic matrices is difficult, and the interfaces between the nanomaterials and matrices are weak. Microscope-based analysis was recently used to quantitatively examine these microstructural features and investigate their contributions to the composites’ mechanical, thermal, and electrical properties. The impacts of the microstructure on these properties are discussed in the first section of this review. In the second section, the various microscopic techniques used to study the distribution of carbon nanomaterials in metallic matrices and their interfaces are described.

## Introduction

Carbon-based nanomaterials such as fullerenes, carbon nanotubes, and graphene are considered keys to overcome the current limitations of conventional materials. Carbon nanomaterials have extraordinary properties and stable molecular structures induced by strong sp^2^ C-C bonds (Phiri et al. 2018; Scarpa et al. 2009). Considerable efforts have been made to increase the specific stiffness, strength, thermal conductivity, and electrical conductivity of metallic matrices by incorporating carbon nanomaterials. However, progress in developing applications for composites has been limited by technical bottlenecks, including poor dispersion of carbon nanomaterials in the metallic matrices and weak interfacial interactions (Choi et al. 2012; Kim et al. 2017).

Many researchers have attempted to resolve the dispersion issue and improve the interfacial properties of these composites by using liquid-phase (Bakshi et al. 2009; Bakshi et al. 2008; Keshri et al. 2009; Goh et al. 2008; Paramsothy et al. 2009; Goh et al. 2006; Uozumi et al. 2008; Laha et al. 2009; Pérez-Bustamante et al. 2009; Esawi and Borady 2008; Esawi et al. 2009) and solid-state processes (Choi et al. 2008; Zhong et al. 2003; George et al. 2005; Choi et al. 2009; Esawi and Morsi 2007; Kwon et al. 2009; Sridhar and Narayanan 2009; Morsi et al. 2010). Liquid-phase processes confer the benefits of cost-effectiveness and the potential for upscaling. However, it is very difficult to disperse carbon nanomaterials in liquid metals because the nanomaterials are initially entangled or agglomerated due to van der Waals forces. Layered coating processes such as plasma spraying (Bakshi et al. 2009), cold spraying (Bakshi et al. 2008), and thermal spraying (Keshri et al. 2009) have been proposed to improve the dispersion of carbon nanomaterials in liquid-phase processes to produce bulk composites. Casting with high-speed mechanical or magnetic stirring tools has also been shown to facilitate the dispersion of carbon nanomaterials in liquid metals (Goh et al. 2008; Paramsothy et al. 2009; Goh et al. 2006; Uozumi et al. 2008). However, poor dispersion and the unwanted transformation of carbon nanomaterials to carbides remain critical drawbacks of liquid-based techniques. Although it has been suggested that the formation of small amounts of Al_4_C_3_ at the interface may enhance interfacial bonding (Laha et al. 2009; Pérez-Bustamante et al. 2009), most researchers have concluded that transforming nanomaterials into carbides degrades the composites’ properties (Esawi and Borady 2008; Esawi et al. 2009).

The relatively low processing temperatures of solid-state techniques are highly advantageous because they prevent unexpected reactions and form fine microstructures. Powder metallurgy techniques that use ball milling are considered effective for the mechanical dispersion of carbon nanomaterials (Choi et al. 2008; Zhong et al. 2003; George et al. 2005; Choi et al. 2009). A metal powder is blended with a carbon nanomaterial, and the composite powder is consolidated through a thermo-mechanical process. Friction stirring processes such as friction-stir welding are increasingly used for solid-state joining and microstructural modification (Esawi and Morsi 2007; Kwon et al. 2009). Heating due to friction and high levels of strain induced during these processes enable microstructural refinement, densification, and the uniform dispersion of carbon nanomaterials. The solution-based synthesis of metal/nano-C powders (Sridhar and Narayanan 2009) and severe plastic deformation (Morsi et al. 2010) have been proposed to improve the composites’ mechanical performance. However, dispersing carbon nanomaterials using solid-state processes remains difficult, and severe mechanical working processes sometimes occasionally destroy the nanomaterials’ molecular structures (Zhong et al. 2003). Poor structuring at the interfaces between the nanomaterials and matrices due to negligible wettability is also consistently reported.

Despite ongoing efforts, the fabrication of metallic matrix composites with uniformly dispersed carbon nanomaterials that form tight interfaces with the matrices remains a challenge. The development of suitable processes is also impeded by the lack of characterization methods to assess their feasibility from a microstructural perspective. It is very difficult to examine carbon nanomaterials in metallic matrices, and methods of systematically and quantitatively analyzing the uniformity of carbon nanomaterial dispersions and interfacial tightness are very limited. In this review, we discuss the impacts of interfacial features and carbon nanomaterial dispersion on the mechanical, thermal, and electrical properties of metallic matrix composites. We also introduce methods used to evaluate the microstructural features of metallic matrix composites that contain carbon nanomaterials. Analysis of these features is needed to better understand the relationships between the processes, microstructures, and properties of the composites.

### Effects of microstructure on the properties of metal/nano-C composites

**Fig. 1:**
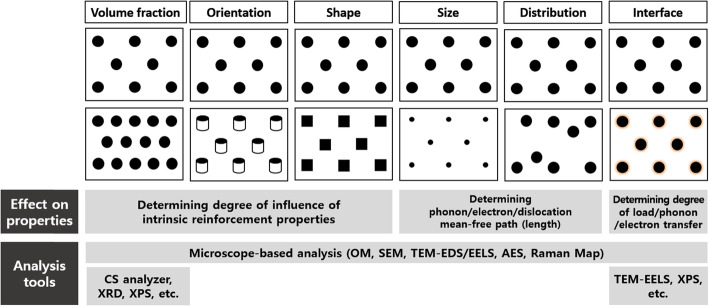
Overview of characterization tools to examine microstructural valuables and their roles on the properties of composites containing carbon nanomaterials

Figure 1 shows important microstructural parameters, their roles in properties, and corresponding analysis tools for composites containing carbon nanomaterials. In composite materials, volume fraction, orientation, shape, size, distribution, and interface with the reinforcement matrix are well-known microstructural parameters. The properties of composites are basically controlled by the intrinsic properties and volume fraction of each phase. The orientation and shape of the reinforcement may determine the degree of influence of the intrinsic reinforcement properties at a fixed volume fraction. For example, the elastic modulus and yield strength of composites follow a simple rule of mixture when continuous reinforcement is perfectly oriented to the loading direction, while the strengthening efficiency decreases if the reinforcement is not aligned to the loading direction. At a fixed volume fraction, the size and distribution of the reinforcement control the distance among the reinforcement, which determines the mean free path of phonons, electrons, or dislocations. The interface is also an important microstructural variable that affects the degree of energy transfer. The load, phonons, and electrons can be readily transferred from the matrix to the reinforcement without energy loss when the matrix and reinforcement have a tight interface.

When the type and volume fraction of the reinforcement are determined, the distribution and interface are two important microstructural features that can be controlled by manipulating the process routes. Analyzing the interfacial features of composites to predict their properties is relatively straightforward. The interfacial region is considered a separate phase with properties that are distinct from those of the matrix and reinforcement. Equation coefficients are occasionally used to determine the scattering of electrons, phonons, or mechanical energy at the interface. The reinforcement distribution is rather difficult to quantitate using theoretical models. Some researchers have attempted to describe the distribution of reinforcements by using the reinforcement distribution coefficient (*α*) (Torigoe et al. 2003). The number of reinforcements per unit area (*x*_*i*_) is used to calculate the coefficient of variation (*ϕ(x)*) with eq. (1).

1 $$ \upphi (x)=\sqrt{\frac{\sum {\left({x}_i-\overline{x}\right)}^2}{n}}/\overline{X}, $$

where $\overline{x}$ and $\overline{X}$ are the average number of reinforcements per unit area and the total area, respectively, and *n* is the number of unit areas. The reinforcement distribution coefficient can be calculated using eq. (2).

2 $$ \alpha =\exp \left[-\upphi (x)\right] $$

Hence, the closer the coefficient *α* is to one, the more uniformly the reinforcements are distributed. The reinforcement distribution can be used quantitatively to predict the mechanical, thermal, and electrical properties of a composite.

The properties of the individual phases in a composite and their volume fractions in the mixture are used to predict the composite’s strength.

3 $$ {\sigma}_c={\sigma}_m{V}_m+{\sigma}_r{V}_r, $$

where *σ*_*c*_, *σ*_*m*_, and *σ*_*r*_ represent the strengths of the composite, matrix, and reinforcement, respectively. *V*_*m*_ and *V*_*r*_ are the volume fractions of the matrix and reinforcement, respectively.

The thermal and electrical conductivities of a composite (*λ*_c_) can be calculated in terms of a mixture by using the two-phase parallel model (Eq. (4)), the two-phase serial model (Eq. (5)), the two-phase serial-parallel model (Eq. (6)), or the Maxwell model (Eq. (7)) (Liu et al. 2017).

4 $$ {\lambda}_{FRC}={V}_{CC}{\lambda}_{CC}+{V}_F{\lambda}_F $$

5 $$ {\lambda}_{FRC}=\frac{1}{V_{CC}/{\lambda}_{CC}+{V}_F/{\lambda}_F} $$

6 $$ {\lambda}_{FRC}=\left(1-{\alpha}_F^2\right){\lambda}_{CC}+\frac{\alpha_F^2{\lambda}_{CC}{\lambda}_F}{\alpha_F{\lambda}_{CC}+\left(1-{\alpha}_F\right){\lambda}_F} $$

7 $$ {\lambda}_{FRC}={\lambda}_{CC}\frac{2{\lambda}_{CC}+{\lambda}_F-2\left({\lambda}_{CC}-{\lambda}_F\right){V}_F}{2{\lambda}_{CC}+{\lambda}_F+\left({\lambda}_{CC}-{\lambda}_F\right){V}_F} $$

Halpin and Kardos modified these models to account for the filler geometry and loading conditions (Halpin and Kardos 1976). Ngo et al. (Ngo et al. 2017) suggested a correction factor of 0.5 to 5 to account for other relevant effects such as the size and distribution of the reinforcements. A weakly conducting interface can be modeled using a standard algorithm to describe the interactions between the matrix and the reinforcements. A thermal resistance value is defined to eliminate phonon and electron flux at the interface (Tian et al. 2019).

**Fig. 2:**
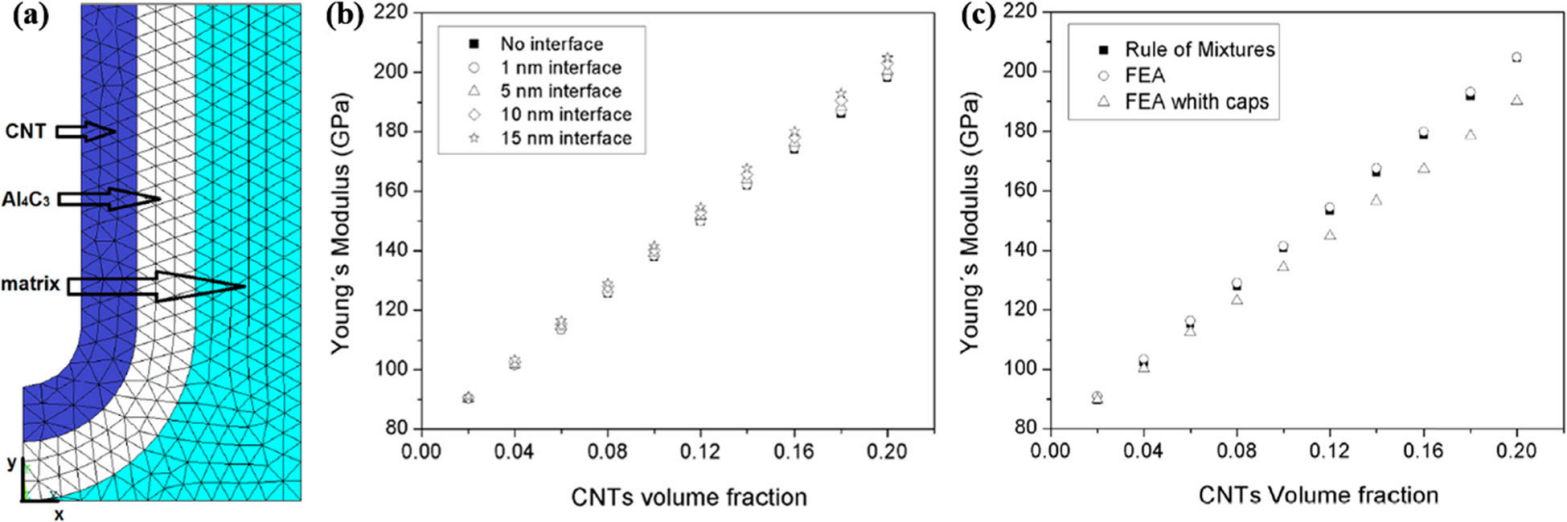
(**a**) Axi-symmetric meshing for the MMC reinforced with 0.20 volume fraction of CNT and interfaces of 15 nm with end-cap, (**b**) Young’s moduli variation vs CNT volume fraction, depending on the thickness of the interfacial Al_4_C_3_ layer for estimations obtained using FEA, and (**c**) Young’s moduli variation vs CNT volume fraction, for estimations obtained using Rule of Mixtures and FEA models, and interfacial Al_4_C_3_ layer thickness of the 15 nm (Alfonso et al. 2015). Reprinted from Alfonso et al. (2015) (*Compos. Struct.* 127, 420–425) with *Composite Structures*’s permission

Predicting the mechanical properties of composites is relatively complex. Composites that contain discontinuous reinforcements such as carbon nanomaterials are thought to exhibit various strengthening mechanisms. For example, a composite may be directly strengthened by load transfer from the matrix to the reinforcements (Dai et al. 2001; Cox 1952; Kamiński 2009), while dislocation can indirectly strengthen it (Zhang and Chen 2006; Vogt et al. 2009; Clyne and Withers 1993; Hazzledine 1992; Huang et al. 1996; Thilly et al. 2001; Arsenault and Shi 1986; Miller and Humphreys 1991; Fleck et al. 2003). Various continuum mechanics models have been suggested over the past several decades to explain load transfer behavior. These include the shear-lag model (Tian et al. 2019; Dai et al. 2001) and the homogenization method (Tian et al. 2019; Cox 1952). The shear-lag model, which involves load transfer through interfacial shear stress, was developed to predict the strength of composites that contain discontinuous reinforcements. Thus, this is the preferred model for discontinuous reinforcements with high aspect ratios. The shear-lag model basically assumes perfect wetting at the interface between a reinforcement and the matrix; hence, energy consumption at the interface is negligible. Using this model, the composite strength can be expressed as (Courtney 2005)

8 $$ {\sigma}_c={\sigma}_m\left[1+\frac{\left(l+{D}_f\right)S}{4l}\right]{V}_f+{\sigma}_m{V}_{m.,} $$

where *l* is the length of a discontinuous reinforcement perpendicular to the applied stress, *D*_*f*_ is the diameter of the discontinuous reinforcement, *S* is the aspect ratio of the discontinuous reinforcement (*l/D*_*f*_), and *V*_*f*_ is the volume fraction of the discontinuous reinforcement. Clearly, the orientations and aspect ratios of discontinuous reinforcements significantly affect the strengths of these composites. Figure 2 shows Young’s modulus calculated on the basis of finite element analysis (FEA) for Al/CNT composites, wherein the effect of the thickness of the interface layer (i.e., A_4_C_3_) and the shape of the end-cap of the CNTs are considered (Alfonso et al. 2015). As indicated, both the interface layer and shape of the reinforcement have a considerable effect on the load transfer efficiency.

Indirect strengthening is well established in the literature. Reinforcements can further contribute to matrix strengthening by forcing dislocation activity to bypass the reinforcements, which is known as Orowan strengthening (Kamiński 2009; Zhang and Chen 2006; Vogt et al. 2009; Clyne and Withers 1993; Hazzledine 1992; Huang et al. 1996). Thermal mismatch strengthening occurs when geometrically necessary dislocations are induced by thermal mismatch between the matrix and the reinforcements (Thilly et al. 2001; Arsenault and Shi 1986). However, these models are valid only when the matrix undergoes conventional plastic deformation as would a coarse-grained metal.

Because of the presence of dispersed nano-scale reinforcements in the matrix, dislocation loops form as dislocation lines and bypass the reinforcements. An increase in strength (*Δσ*_*ORW*_) due to interactions between the dislocations and reinforcements is predicted by the Orowan mechanism (Orowan 1934):

9 $$ {\Delta \sigma}_{ORW}=\frac{E_mb{V}_f^{\left(1/2\right)}}{r\ln \left({D}_f/{r}_0\right)}, $$

where *E*_*m*_ is the Young’s modulus of the matrix, *r* is the spacing between reinforcements, and *r*_*0*_ is the core radius of dislocation. More uniformly dispersed carbon nanomaterials thus have smaller inter-reinforcement spacing (*r*), which strengthens the composite. However, Orowan strengthening is appreciable only when the grains in the matrix are much larger than the reinforcements. Furthermore, because reinforcements often lie on grain boundaries in the matrix, it is unclear whether the Orowan mechanism is possible under these circumstances.

Residual thermal stress induces geometrically necessary dislocations at the interface between a reinforcement and the matrix, which increases the level of flow stress. The increase in strength due to thermal mismatch strain (*Δσ*_*CTE*_) can be expressed by (Luster et al. 1993)

10 $$ \Delta {\sigma}_{CTE}=\alpha Gb{\rho}^{\left(1/2\right)},\kern0.5em $$

where *α* is a constant value of 1.25 and *ρ* is the dislocation density at the interface between a reinforcement and the matrix. Dislocations generated by thermal mismatch strain can generally be removed with a recovery process such as annealing. Coefficients in the theoretical models used to predict the mechanical properties generally reflect inhomogeneous distributions and weak interfaces, and similar models are used to predict the thermal and electrical properties.

### Investigation of metal/nano-C composite microstructures

#### Distribution of carbon nanomaterials

Microstructural features can be analyzed using a variety of microscope-based characterization techniques as summarized in Fig. 1. The volume fraction of the reinforcement in the matrix is quantitatively measured using a carbon/sulfur (CS) analyzer, X-ray diffraction (XRD), X-ray photoluminescence spectroscopy (XPS), and other methods. Chemical bonds at the interface between the reinforcement and the matrix can be investigated using transmission electron microscopy (TEM) combined with electron energy loss spectroscopy (EELS) and XPS.

**Fig. 3:**
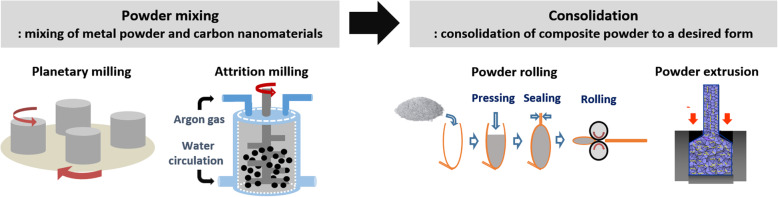
Typical powder metallurgical routes to produce metal matrix composites containing carbon nanomaterials

Here, we first discuss analysis techniques to examine the distribution of carbon nanomaterials. Powder-based technology has recently been used to improve the dispersion of carbon nanomaterials in metal/nano-C composites. A powder process consists of two important steps as described in Fig. 3. A carbon nanomaterial is first mixed with a metallic powder by hand or with a mechanical device such as a blender or ball mill. The mixture is then consolidated and sintered to produce a bulk composite. Hence, the distribution of the carbon nanomaterial in the composite powder and final bulk composite can be evaluated at each step.

**Fig. 4:**
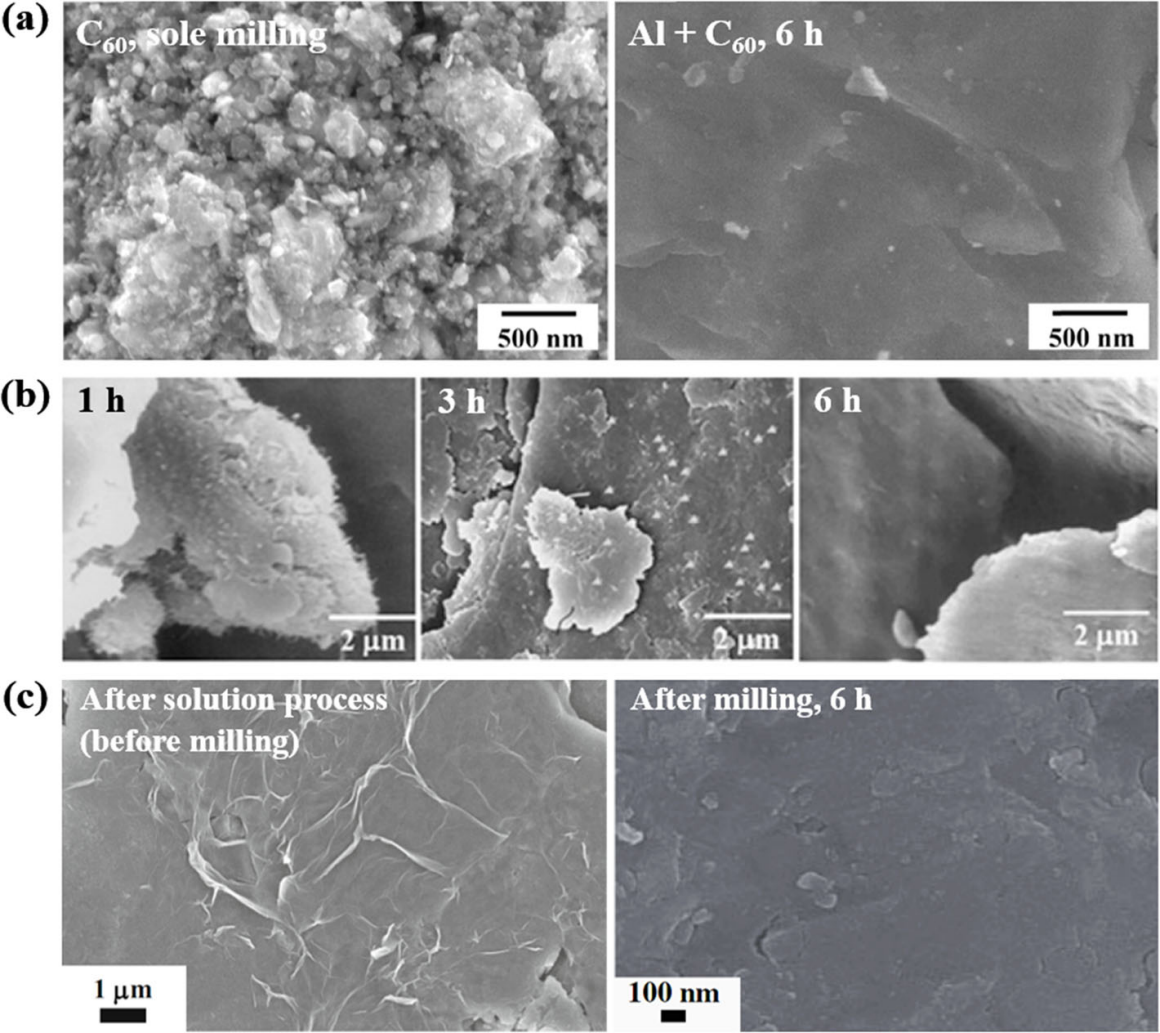
SEM images of (**a**) Al/fullerenes (Choi 2013), (**b**) Al/CNTs (Choi et al. 2009), and (**c**) Al/graphene composite powders, observed at different milling stages (Kim et al. 2017; Kim et al. 2018). Reprinted from Choi (2013), Choi et al. (2009), Kim et al. (2017) and Kim et al. (2018) (*Compos. Res.* 26, 111–115, *J. Mater. Res.* 24, 2610–2616, *J. Mater. Sci.* 52, 12,001–12,012 and *J. Compos. Mater.* 52, 3015–3025) with *Composites Research*’s, *Journal of Materials Research*’s, *Journal of Materials Science*’s and *Journal of composite Materials*’s permission

Scanning electron microscopy (SEM) is generally used to evaluate the distribution of carbon nanomaterials in composite powders. Because the electrical conductivities of metal/nano-C powders are typically poor, the powders are coated with platinum (Pt) to create a conductive surface to facilitate imaging. SEM images of various ball-milled composite powders are shown in Fig. 4. The fullerenes in Fig. 4a were obtained by first disintegrating fullerene aggregates in ethyl alcohol to weaken the van der Waals interactions between the molecules. The fullerenes were then distributed in aluminum powder by attrition milling (Choi 2013). Although the individual fullerene molecules were approximately 1 nm in diameter, the molecules aggregated during the milling step to form giant particles of ~ 200 μm in diameter. The giant particles exhibited the long-range periodicity of a face-centered cubic (FCC) crystalline structure. Figure 4b shows carbon nanotubes dispersed in aluminum powder. In the early stages of milling, the nanotubes were mostly located on the surface of the powder. With three hours of additional milling, the hard carbon nanotubes became embedded in the soft aluminum powder and were gradually dispersed due to plastic deformation of the powder. After six hours of milling, the carbon nanotubes were fully embedded in the aluminum powder and were no longer visible in the SEM images. It is more difficult to disperse graphene in metal powders because of their two-dimensional morphology. Solution processes are frequently used to disperse graphene in aluminum powder prior to milling. Aluminum powder is occasionally flattened before a solution process to increase its specific surface area and transform its gross morphology from that of spherical particulates into flakes resembling graphene. The flaky aluminum powder can then be coated with graphene oxide by mechanically stirring the compounds in an aqueous polyvinyl alcohol (PVA) solution. In this step, hydroxyl functional groups are introduced into a thin aluminum oxide film on the Al surface. The hydroxyl groups can form chemical bonds with functional groups in graphene oxide such as hydroxyl, carboxyl, carbonyl, and epoxy groups (Kim et al. 2017). Graphene oxide can also be reduced to obtain reduced graphene oxide (rGO), and the rGO is further dispersed in aluminum powder via mechanical milling.

Although SEM images reveal the morphologies, locations, and distributions of carbon nanomaterials, changes in their molecular structures during fabrication should be monitored by Raman analysis. The Raman spectra of carbon nanomaterials typically contain the G-band characteristics of graphite and the D-band, which arises from defects. When carbon nanomaterials are damaged or deformed during a process, the D-band and G-band shift to higher wavenumbers. Peak shifts to higher wavenumbers in the Raman spectra of ball-milled specimens may arise from compressive forces in the nanomaterials imparted by the high-velocity impact of the balls. Changes in the interatomic distances between carbon atoms also cause peaks to shift to higher wavenumbers (Choi et al. 2012). The severity of collisions between the milling balls and the powder may generate numerous defects, and the intensity of the D-band of ball-milled specimens may exceed that of the G-band.

**Fig. 5:**
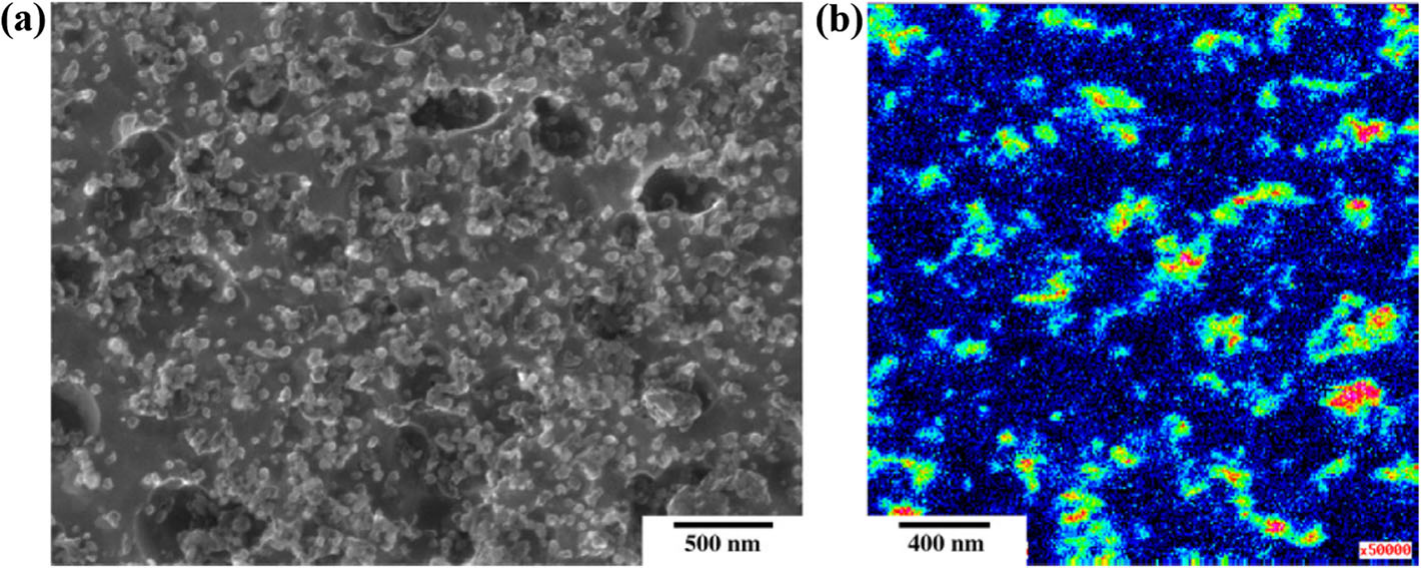
(**a**) SEM micrograph and (**b**) AES map for carbon, obtained from friction-stir processed Al/CNTs composites (Izadi and Gerlich 2012). Reprinted from Izadi and Gerlich (2012) (Carbon 50, 4744–4749) with *Carbon*’s permission

Carbon nanomaterials embedded in the final bulk composite can be examined in SEM images by etching the matrix material with an appropriate acid, as shown in Fig. 5a. Because the interface between the carbon nanomaterial and the metallic matrix has higher energy than the matrix, it will be etched much more quickly than the matrix to reveal the potential location of the nanomaterial. Auger electron spectroscopy (AES) can be performed along with SEM analysis. The AES elemental map in Fig. 5b shows the distribution of the carbon-rich phase in an Al/CNT composite. The volume fraction of this secondary phase can be determined using image analysis software. Compared to energy-dispersive X-ray spectroscopy (EDS) mapping, AES is considered more suitable for analyzing the distribution of carbon nanomaterials; AES enables nanoscale compositional analysis due to the relatively short mean free path of the Auger electrons on the order of a few nanometers compared to those of X-rays. However, this means that only those produced near the surface layer can escape to the free space to be collected. Hence, the specimen should be ultra-thin for the AES analysis. In-house indentation can be performed to compare the hardness of carbon nanomaterials to that of the metallic matrix (Izadi and Gerlich 2012).

**Fig. 6:**
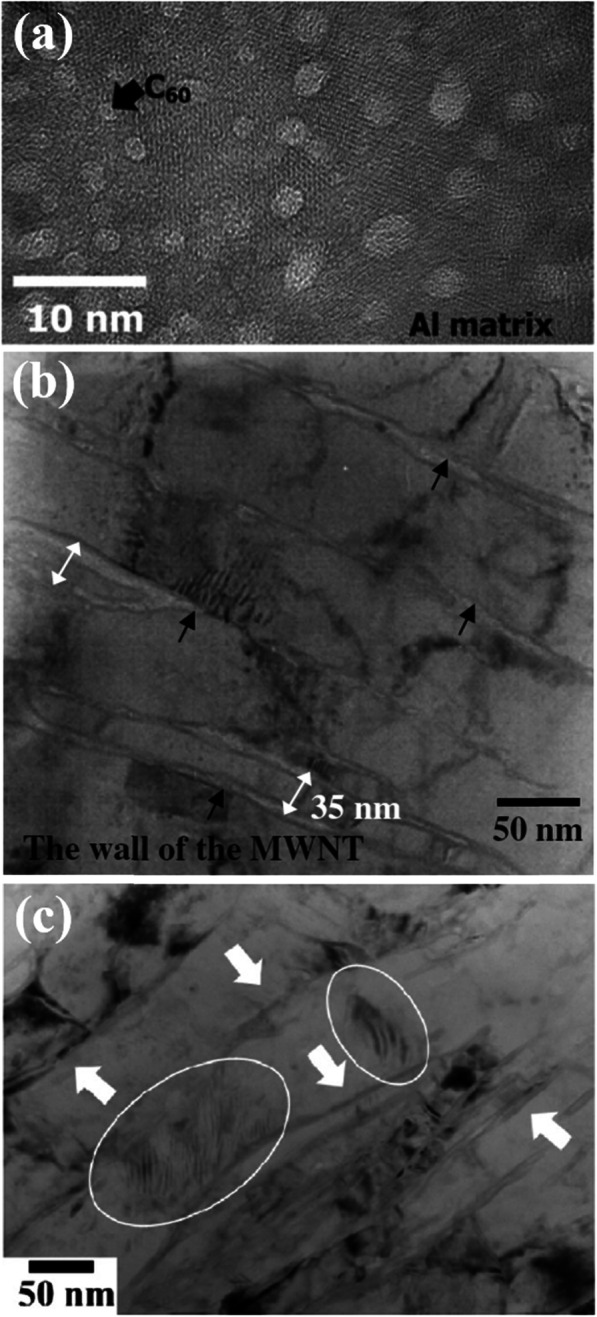
HR-TEM images of (**a**) Al/fullerenes (Choi et al. 2010), (**b**) Al/CNTs (Choi et al. 2008), and (**c**) Al/graphene composites (Shin et al. 2015a). Reprinted from Choi et al. (2010), Choi et al. (2008), and Shin et al. (2015a) (*Carbon* 48, 3700–3707, *Scr. Mater.* 59, 360–363, and *Carbon* 82, 143–151) with *Carbon*’s and *Scripta Materialia*’s permission

TEM is the most popular method to examine the distribution of carbon nanomaterials. Bright field (BF)-TEM images of various composites are shown in Fig. 6. The composites contain fullerenes (Fig. 6a), carbon nanotubes (Fig. 6b), and graphene (Fig. 6c). Because carbon nanomaterials are lighter than metallic matrices, they appear brighter in BF-TEM images. High-resolution (HR)-TEM imaging enables examination of the molecular structures of carbon nanomaterials, typically graphitic fringes, and interfacial structures within the metallic matrices. The transformation of carbon nanomaterials into carbides can also be detected using HR-TEM imaging and corresponding diffraction pattern analysis. These transformations are difficult to detect through XRD analysis due to the small size and volume. The formation of aluminum carbides during the fabrication of Al/C composites with carbon fibers or carbon nanotubes is frequently reported. This is attributed to the relatively low free energy of aluminum carbide formation, which is − 12.7 kcal at 298 K (Park et al. 1994). The formation of nanoscale Al_4_C_3_ with a fringe spacing of 0.84 nm in the (001) plane is often observed.

#### Investigation of interfacial features

**Fig. 7:**
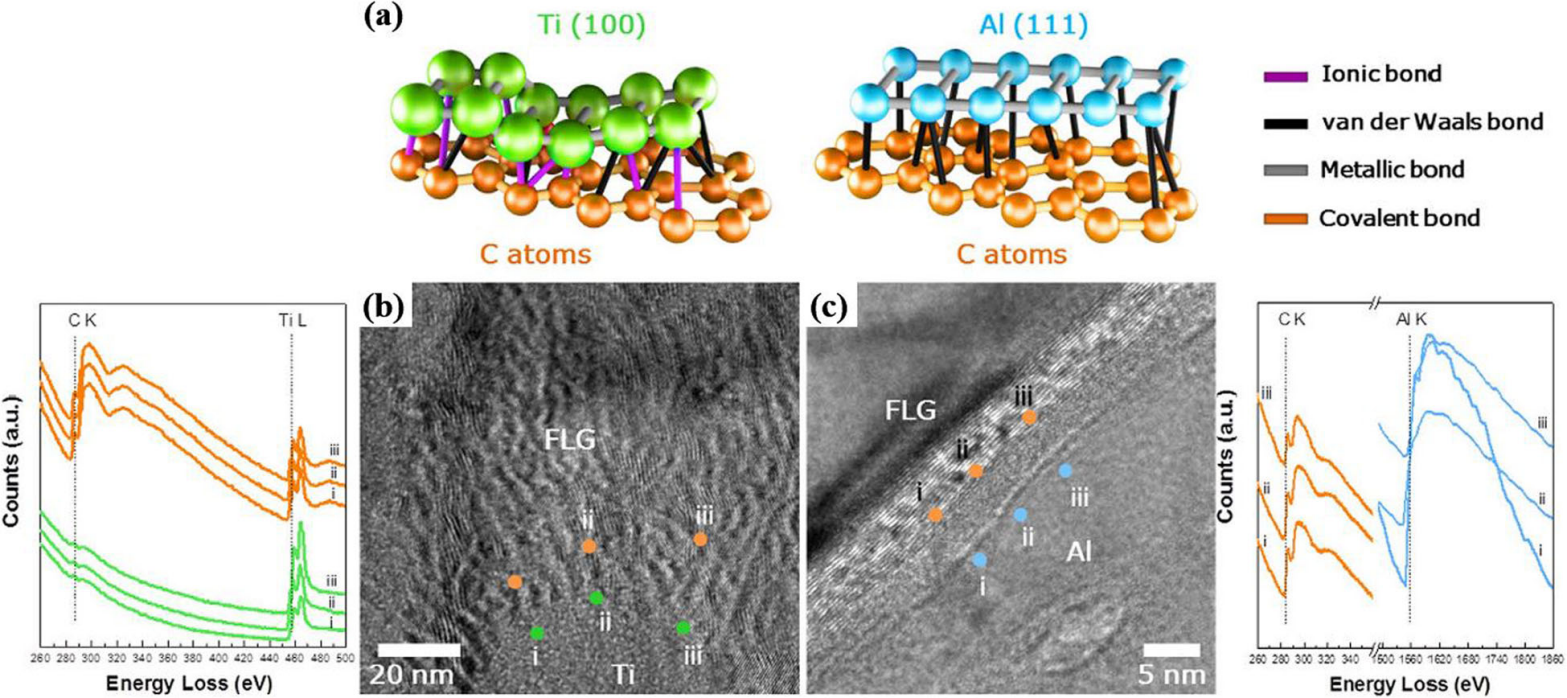
(**a**) Schematic of bonding features for FLG/Al and FLG/Ti composites. HRTEM images of (**b**) FLG/Ti and (**c**) FLG/Al composites and their corresponding EELS spectra taken from the FLG/Ti and FLG/Al composites (Shin et al. 2015b). Reprinted from Shin et al. (2015b) (*Sci. Rep.* 5, 16,114) with *Scientific Report*’s permission

Interfacial structures can be analyzed using TEM in combination with EELS. The bonding features of Al/graphene composites and Ti/graphene composites are compared in Fig. 7a. Energetically favorable adsorption sites for Al and Ti atoms in the graphitic structure can be predicted using density functional theory (DFT) simulations. Carbon atoms in the basal graphitic plane are joined together by strong covalent bonds. The remaining *p*_*z*_ orbitals allow the carbon atoms to bond with metals outside the plane. Nontransition metals (such as Al) form weak secondary bonds with graphene because they lack *d*-orbital subshells and have a very limited affinity for carbon. However, transition metals such as Ti have unfilled *d*-orbitals. Electrons in *d*-orbitals can participate in ionic bonds with dangling carbon atoms on graphene. Calculations have revealed that overall bonding between the basal plane of Ti and a single layer of graphene is approximately five times stronger than bonding between Al and carbon (Shin et al. 2015b).

Observation of composites at the atomic scale can yield important information about their interfacial structures. The interface between graphene and the Ti matrix in Fig. 7b differs from that between graphene and the Al matrix in Fig. 7c. The HR-TEM image of the Al/graphene composite shows typical lattice fringes of a single graphite layer with an interlayer spacing of ~ 0.34 nm. These lattice fringes are not visible in the HR-TEM image of the graphene/Ti composite. The differences between the bonding features of the two composites can be examined in more detail using EELS. Slight variations in the EELS spectra corresponding to points (i)–(iii) in the HR-TEM images indicate the presence of partially balanced, incomplete metal-carbon bonds in both composites. Carbon in graphene typically produces a peak at 285 eV, while Al in the Al matrix generates a peak at 1563 eV. Ionic Al-C bonds will generate Al and C peaks at 73.4 eV and 282.2 eV, respectively; therefore, they are not indicated near the interface. As a transition metal, Ti is strongly electrophilic and reacts to form ionic Ti-C bonds. Thus, Ti participating in ionic Ti-C bonds in few-layer graphene (FLG) composites generates a high-intensity peak at 458 eV. The results can be confirmed with XPS, which enables an analysis over larger areas.

**Fig. 8:**
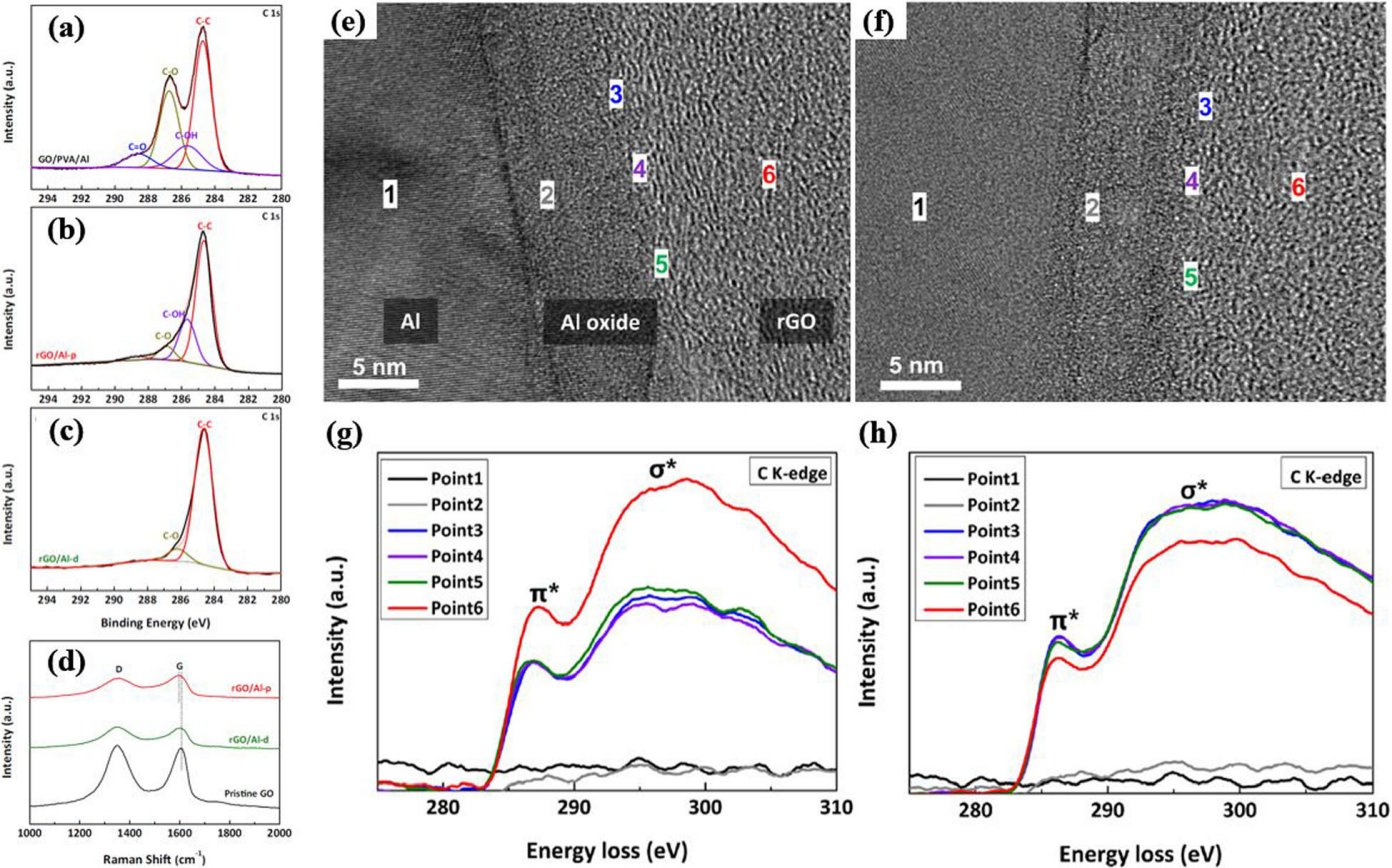
XPS analysis of (**a**) GO/PVA/Al, (**b**) rGO/Al-p, (**c**) and rGO/Al-d hybrid materials together with (**d**) Raman spectra of pristine GO (black line), rGO/Al-d (green line) and rGO/Al-p (red line). (**e**), (f) HRTEM images of rGO/Al-p and rGO/Al-d together with (**g**), (**h**) their corresponding EELS spectra acquired from the marked points ‘1–6’in (**e**) and (**f**) (Jang et al. 2017). Reprinted from Jang et al. (2017) (*Appl. Surf. Sci.* 407, 1–7) with *Applied Surface Science*’s permission

Figure 8 introduces an example of utilizing XPS, Raman, and EELS analyses to examine the interfacial features between aluminum and rGO (Jang et al. 2017). The authors used PVA to enhance the interfacial bonding between aluminum and rGO by generating a large number of hydroxyl groups on the surface of aluminum plates. By analyzing the position and intensity of the peaks in the XPS spectra, the type and degree of chemical bonds at the interface (for example, epoxy (C-O at 286.6 eV), carbonyl (C=O at 288.2 eV), C-C at 284.6 eV, etc.) was compared for GO/PVA/Al, rGO/Al-p (obtained after heat-treatment of GO/PVA/Al), and rGO/Al-d (an Al plate directly coated with rGO) samples. Furthermore, the red-shift in rGO/Al-p in the Raman spectra can be used as evidence of strong chemical bonds between rGO and aluminum because it may originate from the in-plane tensile strain created during the reaction between the hydroxyl groups on the PVA-modified Al surface and the functional groups on the graphene oxide surface. The authors compared the ratio of the intensity of peaks at 285 eV (corresponding to transitions from 1 s to π* states) and 291 eV (corresponding to transitions from 1 s to σ* states) in EELS spectra acquired at the interface of rGO/Al-p and rGO/Al-d samples. The π*/π* + σ* intensity ratio represents the relative amount of sp^2^ bonds, which also demonstrates that numerous conjugations formed at the interface between aluminum and rGO because of the PVA treatment.

## Conclusions

In metal matrix composites containing carbon nanomaterials, characterization of the carbon reinforcement is quite challenging because carbon is a light element and the size of the reinforced materials is limited to the nanometer scale. In this review, the following microscopic techniques used to examine the dispersion of carbon nanomaterials in metallic matrix composites and their interfacial features were described: SEM, AES, HRTEM, EELS, and XPS. In addition, the effects of the microstructural features on the composite properties were discussed. The deterioration of electrical, thermal, and mechanical properties due to the inhomogeneous distribution of carbon nanomaterials can be predicted using theoretical models by incorporating a distribution parameter for the dispersion of nanomaterials in a mixture. Weak interfaces scatter electrons, phonons, and mechanical energy, which reduce the reinforcing effects of carbon nanomaterials. This reduction can be quantified using a coefficient or by treating the interfacial areas as secondary phases with unique properties. Control of these composites’ microstructures can significantly improve their electrical, thermal, and mechanical properties. Several microscopic analysis techniques used to examine the dispersion of carbon nanomaterials in powders, bulk composites, and the interfacial characteristics of the composites were introduced. These characterization methods can enable the feasibility of a process to be evaluated in microstructural terms. This can facilitate the optimization of process conditions to obtain composites with desirable microstructures and properties.

## Data Availability

Not applicable.

## Abbreviations

AES: Auger electron spectroscopy
CS: Carbon/sulfur
DFT: Density functional theory
EDS: Energy-dispersive X-ray spectroscopy
EELS: Electron energy loss spectroscopy
FCC: Face-centered cubic
FEA: Finite element analysis
FLG: Few-layer graphene
HR-TEM: High-resolution TEM
Pt: Platinum
PVA: Polyvinyl alcohol
rGO: Reduced graphene oxide
SEM: Scanning electron microscopy
STEM: Scanning TEM
TEM: Transmission electron microscopy
XPS: X-ray photoluminescence spectroscopy
XRD: X-ray diffraction
